# What Is the Role of Imagined Futures in the Development of E-Cigarette Use among Young People?

**DOI:** 10.1177/00380385241290745

**Published:** 2024-10-23

**Authors:** Jason Hughes, Kahryn Hughes, Grace Sykes, Michelle O’Reilly, Charlie Sutton, John Goodwin, Khalid Karim

**Affiliations:** University of Leicester, UK; University of Leeds, UK; University of Leicester, UK; University of Leicester, UK; Loughborough University, UK; University of Leicester, UK; University of Leicester, UK

**Keywords:** adolescents, e-cigarettes, futures, gateway theory, investments, smoking, trajectories, vaping

## Abstract

Public health ‘gateway’ narratives concerning young people’s e-cigarette use warn against a future generation beset by escalating addiction and a possible epidemic of tobacco- and vaping-related illnesses. We argue that such imaginaries of vaping futures are in fact based in smoking pasts, which, while likely not ‘real’ in the sense of predicting the development of youth vaping, have real consequences through influencing the conditions of young people’s e-cigarette use. Drawing on a study of 14–18-year-old vapers, we consider how the future imaginaries of gateway thinking – characterised by escalating dependence – both oppose and intersect with a cultural stock of neo-liberal future imaginaries – marked by progressive independence and self-determination. We show how both sets of imaginaries are negotiated and entangled within the logics and practices of young vapers’ ‘futures-in-process’ to advance debates in the sociology of futures and offer a radical rethinking of substance use by youth.

## Introduction

A concern with futures is arguably a defining one for sociology (Adam, 2004). Indeed, classical sociologists’ preoccupation with transformative social change and with how certain imagined futures might variously be facilitated, resisted, constrained and/or enabled has received renewed attention through more explicit considerations of ‘futures thinking’ and ‘futures making’ (Halford and Southerton, 2023) (e.g. Adam, 2004, 2011, 2023; Oomen et al., 2021; Tutton, 2017). However, conceptual scholarship on futures and more empirical applications have, with some notable exceptions, tended to emerge as parallel sociological developments that rarely ‘speak to one another’ (Gokmenoglu, 2022: 646).

This article advances debates in this area by developing a dialogue between theoretical and more empirically driven considerations of temporality and futures using the case of e-cigarette use by young people, particularly the concern that youth vaping might serve as a ‘gateway’ to smoking and the use of other substances. In doing so, we seek not only to contribute to the sociology of futures, but to more general policy and scientific debates relating to substance use by young people, which, we shall show, invariably centre on a particular anticipatory framing of prospective individual and generational futures.

Our empirical data draw from a Cancer Research UK (CRUK)-funded study of a diverse sample of young people in Leicestershire, UK entitled ‘Adolescent Vaping Careers’ (C60744/A23882). Using both retrospective and prospective narrative interview methods, we explored the e-cigarette and combustible tobacco use trajectories of a diverse sample of 36 14–18-year-olds. Supported by a qualitative longitudinal methodology, our original study centrally considered the extent to which e-cigarettes might act as a ‘gateway’ to combustible tobacco, and possibly other drugs.

Through this research, we observed that gateway thinking operates less as a precise scientific hypothesis than as a pervasive cultural trope expressing a particular future imaginary relating to young people’s substance use: ‘if they start *here*, it is likely that they’ll end up *there*’. While the ‘there’ is often imprecise and open-ended, this imagined future centrally anticipates escalating risk, harm, dependence and diminishing degrees of self-determination wherein young people who vape nicotine *today* are understood to be more likely to smoke tobacco, and possibly other drugs, *tomorrow*.

Gateway framings underpin major policy discussions concerning the regulation of e-cigarette and tobacco consumption, marketing and material products (e.g. Nkansah-Amankra, 2020). As a case in point, the former UK Conservative government voted in early 2024 to create a ‘smoke-free generation’ by increasing the legal age to buy tobacco by a year, every year, from 2027 onwards (a bill that is set to be reintroduced under the current Labour administration). In debating the motion for a ‘smoke-free future’, MPs discussed further restricting the promotion and marketing of e-cigarettes to young people since, as the Democratic Unionist Ian Paisley put it, ‘children [see] vaping as a gateway into something, and that is very serious’ (*Hansard* HC Deb. vol.743 col.167WH, 11 January 2024).

Significantly, Paisley did not need to articulate what that serious ‘something’ might comprise. It was left to ‘the imaginary’ invoked by the term ‘gateway’. This pervasive anticipatory future-framing characterises more general debates relating to substance use by young people, and has effectively compelled researchers to engage with e-cigarette use temporally. Key here are longitudinal studies that examine the possible sequential relationships between vaping and smoking, particularly among young people (e.g. Nkansah-Amankra, 2020; Pierce et al., 2021). Temporality and imagined futures have therefore become fundamental to policy, practitioner and scientific debates about e-cigarette use such that the intersection of ideas around gateways and concerns about youth are arguably *the* defining topics of much of the research in this area (e.g. King et al., 2020).

Most significantly, the discursive environment through which gateway framings have become widespread is also one in which young people are urged to make sense of their substance-using behaviours in terms of *possible futures*. This is particularly so for adolescents undergoing multiple developmental transitions, and who are recurrently impelled to take account of their future adult selves against the yardstick of neo-liberal imaginaries of late-modern selfhood whereby individuals are understood as *pilots of their own destiny* (Beck, 1992; Giddens, 1991). Such futures are marked by progressive degrees of self-determination and independence – the counterpoint to the cautionary futures imagined through gateway thinking.

These two future imaginaries – one born of protectionist concern with escalating *dependence*, another expressing the neo-liberal ideal of greater *independence* – are variously *real in their consequences* (Thomas and Thomas, 1928) through constituting an oppositional discursive intersection that, we shall argue, has come materially–culturally to pervade the conditions under which young people use e-cigarettes.

Our central concern in this article, then, is to explore how young vapers understood, managed and negotiated such seemingly divergent imagined futures, and in so doing, to consider the significance of such practices for materialising ‘futures-in-process’. Doing so allows us to advance discussions in the sociology of futures by empirically exploring how processes of future orientation and future making are fundamentally interrelated. Moreover, it enables us to highlight how dominant imaginaries of vaping futures are based in smoking pasts, and to propose a radical rethinking of such ‘retrospective anticipatory’ framings as these relate to more general substance use by young people.

## Background: Gateway Futures, Past and Present

### Present Futures and Future Presents

Core sociological debates regarding futures relate to how social analysts might go about researching the ‘not yet’ (Tutton, 2017: 481). Key in these debates is the work of Adam (2004, 2011, 2023), whose arguments are particularly useful for the analyses we present in this article, and upon whose work we will draw extensively. Adam (2004: 10) proposes that earlier sociological attempts to engage with futures directly have typically concerned themselves exclusively with ‘how the future is envisaged’ and how ‘future ideals and utopias guide social actions in the present’. Such preoccupations solely with ‘present futures’, Adam (2023: 286) contests, are typically at the expense of a consideration of collective ‘future presents’ – how ‘the social world of our making . . . affects not just our own future but those of contemporaries across the globe as well as that of successor generation*s*’ (2004: 10).

Adam warns against a ‘disjuncture’ between a future-*oriented* and a future-*producing* sociological subject (Adam, 2023: 280). Her key point is that while future presents are *immaterial*, or perhaps better, not yet material in the sense that they are not directly observable in the present, they are nonetheless in the process of being *materialised*. That is to say, they are ‘processes-in-progress’ entailing a ‘largely latent and in/visible materiality’ (2023: 282). This presents a challenge for sociologists who are concerned with ‘minding’ futures since such processes warrant new approaches, methods and techniques to allow incorporation into social analyses (Adam, 2004).

To advance these discussions, our approach in this article is orientated towards *futures-in-process*, which are not solely ‘immaterial’ nor exclusively ‘of the mind’ (Taylor, 2003: 106), but which, following Tutton (2017), involve a wide range of ‘material–discursive practices’ (Tutton, 2017: 483). An examination of youth vaping is an exemplar in this respect since it involves *simultaneous* processes of both future orientation and future making. To use Adam’s language, we explore how the *present futures* of the young vapers in our study are fundamentally interrelated to their *future presents* (Adam, 2023). More concretely, in examining futures-in-process, we consider the dynamic interplay between young vapers’ imagined and materialising *biographical* futures, and their related *generational* futures. Of particular significance in this respect are gateway-inspired anticipations of a future generation of nicotine addicts beset by tobacco-related and/or as-yet-unknown vaping-related, morbidities.

Importantly, the concerns related to gateway thinking here also serve to exemplify a related problem highlighted by Adam (2023): that models of the future tend to be constructed through evidence from the past, with often simplistic (and typically faulty) unilinear temporal extrapolations. Our core concern in this respect is with how what is known about smoking pasts has had a bearing upon what is anticipated in relation to vaping futures, and, in turn, over the futures-in-process that we have explored in the accounts of young e-cigarette users.

### Gateway Pasts and Futures

Thus far we have purposely referred to ‘gateway thinking’ rather than ‘gateway theory’ because what we refer to involves an array of parallel ideas, not a singular or coherent theory or hypothesis (Bell and Keane, 2014). Indeed, the antecedents of gateway thinking relating to e-cigarette use articulate a long and disparate history. In early-modern England, for instance, leading physicians expressed concern that tobacco users would become ‘dried out’ by the practice and, consequently, more likely to drink alcohol to excess in their efforts to regain humoural equilibrium (see, for example, Hughes, 2003). This association between smoking and drinking was further reinforced through the notion that tobacco users were prone to consume tobacco ‘like Tinkars doe ale’, with tobacco understood by such opponents as a ‘plague, a mischefe, a violent purger of goods, lands, health’ that would render smokers unfit for labour and ultimately lead to the ‘ruine and overthrow of body and soule’ (Alexander, 1930: 89; Burton 1638; Hughes, 2003).

Such past imaginaries highlight several of the hallmarks of ‘gateway’ thinking, albeit without any reference to the term. These, we shall show, endure into future imaginaries of major significance to the young vapers we interviewed. They include, first, the idea that the initial use of one, supposedly less dangerous, substance will lead to the future use of another more harmful one. Second, this anticipated sequential progression defines a future marked by escalating risk and diminishing self-control (Bell and Keane, 2014). Third, relatedly, this future imaginary renders a potentially innocuous substance/practice *guilty by sequential association* with a supposedly more dangerous one.^1^ Accordingly, smoking was understood as ‘bad’ not solely because it was thought to lead to drinking, its posited sequential connection to drinking also prompted *direct* comparisons to alcohol – a paradigmatic compound expressed in such 17th-century terminology of smokers as ‘tobacco drinkers’ and ‘dry drunks’ (Hughes, 2003).

The more specific articulation of ‘gateway’ futures is relatively recent, emerging in US drug policy discourse in the 1930s, with the terminology ‘gateway drug’ adopted from the 1960s onwards. Particularly since the 1980s, the term has become increasingly hegemonic and applied to a widening range of imagined substance and behavioural futures including, most recently, the idea of vaping leading to smoking (Bell and Keane, 2014).

### Smoking Pasts and E-cigarette Futures

Gateway concerns about e-cigarettes relate simultaneously to biographical and generational future imaginaries – both of the substance-using trajectories of individual e-cigarette users, and to a potential future generation of addicts (both to combustible tobacco, and possibly other drugs) lured in by the seeming innocuity of e-cigarettes (Galderisi et al., 2020). Despite societal-level data suggesting a progression in the opposite direction – with the UK now experiencing the lowest levels of teen smoking since records began – the future imaginary of gateway thinking has proven enduring in lay, public health and policy discourse, in particular through the notion that vaping might represent a ‘ticking timebomb’ (Hughes et al., 2021).

While there remains considerable debate over the potential longer-term impact of e-cigarette use on population health, a substantial body of work based on painstaking, independent reviews of the evidence (e.g. analyses of cytotoxicity profiles, metabolomic studies of biomarkers, etc.) suggests that e-cigarettes are significantly safer than tobacco (see, in particular, McNeil et al., 2015). Despite this, recent studies in the UK have found that less than half, 46%, of 11–17-year-olds surveyed believe e-cigarettes to be *less* harmful than combustible cigarettes – a proportion that has steadily decreased, from 73%, over the past decade (ASH, 2023). As our own participants attest, the idea that e-cigarettes *might* be found to be more harmful in the future is easily confounded with their treatment as though they were already *known* to be more harmful. In such ways, we propose, projected generational futures are not solely imaginary and ‘discursive’; they form part of materialising ‘futures-in-process’, here conditioning users’ technologies, practices, identifications, associations and orientations, and directly influencing the broader policy–discursive, social and informational landscapes within which these develop.

Significantly, e-cigarettes are regulated in the EU (since 2014) and the USA (since 2016) as *tobacco* products – a classification that remains much disputed (see, for example, Munafò, 2019). E-cigarettes are a highly heterogenous category, not a singular product, encompassing a wide array of technologies and products that vary by function, content and appearance (Hitchman et al., 2015). Some e-cigarette liquids contain no nicotine; and nicotine itself can be synthesised without tobacco, with products already available, such as PuffBar in the USA, exclusively containing nicotine derived from a synthetic pathway (ethyl nicotinate, an ester of niacin) (Jordt, 2023). Somewhat ironically, then, the category ‘e-cigarette’, through its partly skeuomorphic association with smoking pasts, encompasses a class of ‘tobacco products’, some of which contain no tobacco at all.

In these and other ways, the ‘past futures’ (Adam, 2023: 285) of tobacco inform both the ‘present futures’ and, as we shall show, the materialising ‘future presents’ of e-cigarette use/rs.

## Methods

Our study employed a qualitative longitudinal research design to facilitate a processual engagement with young people’s e-cigarette use. Two phases of interviews were conducted between January 2018 and August 2019, with interviews separated by between six to 12 months. A total of 66 in-depth interviews were conducted, 36 in the first round and 30 in the second (six participants withdrew after moving school/college). This approach supported a modest diachronic interrogation of continuity and change in participants’ understandings, uses and experiences of e-cigarettes and other substances. Additionally, our open-ended questions were designed and structured to elucidate shifting orientations towards past, present and future use, again supporting a concern with temporality and futures.

After securing research ethics approval, we worked with existing partners and contacts to adhere to institutional safeguarding protocols and optimise practices regarding recruitment and access to a sample of 14–18-year-olds who identified as current e-cigarette users (used within the past month), some of whom had used combustible tobacco before, after or concurrent with their vaping. We recruited 36 young people from a diverse cross-section of schools, colleges and youth organisations across Leicester city and Leicestershire. With 59.1% of its population identifying with non-white ethnic groups in the 2021 census, Leicester has considerable ethnic and socio-economic diversity. Our sample reflected this: 18 of the 36 participants identified as belonging to black, Asian and minority ethnic (BAME) groups (nine Asian British Indian, three Asian British Pakistani, one Other Asian British, five Mixed/Multiple), 14 identified as female, 22 as male and roughly 50% were from some of the most socio-economically disadvantaged communities in the UK (Leicester City Council, 2019). Under half of our sample (17) vaped regularly (more than once per week), 12 were frequent users (more than once per day) and the remainder were sporadic experimenters. Thirty participants identified as sole e-cigarette users although, during the interviews, it became clear that many (29) had smoked at least once before (25) or after (four) first having used e-cigarettes.

Our exploratory analyses were multiple and ongoing. They involved our bringing emergent interpretations of the findings into ‘analytic conversation’ (Hughes et al., 2022) between members of the research team, and through an iterative dialogue, with theory, questions, concepts and evidence. Across two phases of analyses, we analysed participants’ accounts of vaping, smoking and other substance use, focusing specifically on anticipatory orientations, practices and narratives.

The following discussion focuses particularly on the contrasts and differences within our sample, including how participants’ accounts (anonymised and pseudonymised below) variously serve to articulate, reframe and counter various future imaginaries. We group our findings into three sections that broadly correspond to the key theoretical foci of our discussion. First, we explore responsibilisation and uncertainty, focusing on the fundamental relationship between smoking pasts and vaping futures in the accounts of participants. Second, we examine participants’ ‘techniques of futuring’ – how the young people in our study negotiate contrasting future imaginaries in their materialising ‘futures-in-process’. Finally, we consider participants’ anticipations of independence and dependence to explore how imagined futures are not solely imagined, nor in any simple sense ‘produced’, but are emergent, materialising, contingent upon ongoing investments that are simultaneously governed by the conditions of their own possibility.

## Findings

### Determining Uncertain Futures: ‘I’ve Done the Research’

Without exception, the young people in our study either implicitly or explicitly expressed the understanding that the responsibility for their futures was largely ‘in their own hands’. In relation to their commencement of vaping, participants typically recounted having undertaken independent research about vaping prior to, or shortly after, having started:

Yeah. I did some research. Back in like Year 7, the shisha pens . . . people used to do that. And my parents, like, knew about it and they were like, ‘Stay away from that.’ Since then, like, I researched it . . . and decided there weren’t that many, like, complications, like proper health complications. So, I was like, ‘Yeah alright I’ll do it.’ (Zev, 14, South Asian)

Significant in Zev’s account is his seeking to establish for himself the relative risks of e-cigarettes, and his unwillingness to accept at face-value the information from his parents. Zev was at pains to determine the risks or otherwise of vaping and decide his own actions accordingly: a push against parental dependence, and a nod towards his esteem for independent self-determination. Indeed, across the data, participants recounted consulting online searches, peers and an array of informational sources to ‘do the research for themselves’ on the relative risks of vaping.

Zev’s sense of having responsibility to understand, and take responsibility for, the risks relating to a particular practice ostensibly accords with a more general neo-liberal centring of youth as an increasingly problematised social category, with adolescence understood to be risky in the dual sense that adolescents are seen to be prone to engage in risk behaviours that may destroy their futures, and that they are ‘at risk’ of a range of psychological and physical harms consequent on their ‘unfledged’, ‘nascent’ developmental liminality (Steinberg, 2010). Such ideas, in turn, further relate to the more general thesis that in late-modernity, traditional expectations of the future collapse as individuals are increasingly compelled to view their own biographies as a reflexive project of the self involving a heightened burden of choice, risk calculation and individual responsibility (e.g. Beck, 1992; Giddens, 1991).

Relatedly, the felt responsibility to *do the research* by Zev, and many of the other young people in our study, is also expressive of considerable uncertainty regarding the potential future risks of e-cigarette use. Many of our participants expressed the understanding that the ‘verdict is out’ on the potential short- and long-term health consequences of e-cigarette use. Regarding the latter, the future imaginary of a ‘ticking timebomb’ proved to be of particular significance. Former Chief Medical Officer, Professor Dame Sally Davies, used this very locution in relation to the possible future represented by ascendant e-cigarette use in 2015, and such ideas were still very much ‘in the air’ during the period in which our data were collected. Popular press headlines oscillated between claims such as ‘Switching to e-cigarettes reduces the amount of cancer-causing tobacco toxins by 97% in just 6 months’ (*Daily Mail*, April 2017) and, only a few months later: ‘E-cigarettes are as dangerous as smoking – just ONE puff could be all it takes to increase the risk of a heart attack’ (*Daily Mail*, August 2017). During our interviews, we discussed headlines like these directly with participants:

I think everyone’s confused because . . . there are so many conflicting headlines . . . Like, nobody really knows, and everyone’s kind of like going backwards and forwards thinking oh no, it’s really unsafe. And people are cutting down, and it’s actually fine and then everyone will start again . . . so I’ve been consciously keeping it at very low levels, so that even if it was worse than smoking, I’ve only ever used it socially. (Evie, 17, White)

Evie’s extract attests to complex and often contradictory informational landscapes regarding the future consequences of e-cigarette use negotiated by the young people in our study. While some participants found the array of information somewhat bewildering, others often would discriminate between different sources: ‘Well, I just read one . . . “They’re just as dangerous as smoking”, but that’s from, like, *The Daily Mail* . . . [laughs]’ (Lily, 16, White).

The uncertain consequentialist future the ticking timebomb imaginary invokes involves a slippage between the idea that e-cigarettes might *lead to* an explosion in cigarette use in the future via gateway effects and the allied idea that vaping might be *like* smoking inasmuch as long-term e-cigarette use, as is the case with tobacco-related illness, might trigger the incubation of diseases – invisible futures-in-the-making – that take several decades to manifest. In these and other ways, ‘vaping presents’ and ‘vaping futures’ are refracted through ‘smoking pasts’. Such retrospective future imaginaries render vaping not simply potentially *guilty by temporal association* with tobacco, but also, through the caution the dramatic spectre of tobacco-related illness provokes, *guilty until proven innocent*.

As we have argued earlier in this article, gateway future imaginaries are, in this key respect, not simply imagined. Rather, they have materialising consequences both through informing policy and regulatory environments, feeding into a cultural climate of uncertainty regarding the possible future consequences of vaping, and prompting participants to feel responsibilised for determining these. Moreover, as indicated by Evie above, such ideas shape young e-cigarette users’ futures-in-process, with possible adjustments to practices, such as vaping less frequently, in more particular contexts, and, as other participants discussed, at lower wattage levels, in response to the ebbs and flows of contrasting and rapidly-shifting popular discourse. Such *techniques of future making* (Hajer and Pelzer, 2018) involve independent modifications to practices and behaviours ‘now’ in anticipation of a possible future ‘then’. However, rather than marking a point of resigned acceptance, it was often *in opposition to* consequentialist future narratives – particularly gateway anticipations of escalating dependence – surrounding e-cigarette use that our participants would articulate and develop their own imaginaries of to where their vaping might lead in the future.

### Techniques of Futuring: ‘Swimming with Sharks’

Our study initially drew upon gateway-predicated questions, centrally investigating whether or not e-cigarette use might lead to smoking – an anticipation of possible pharmacologically dependent futures. However, we soon found that this mode of questioning superimposed a connective logic that was resisted by our participants who instead employed a range of ‘logics of sequential connection’ (see also Hughes et al., 2021) at odds with our own:

I: Has vaping made you want to try other things?

P: I want to do all sorts of things, like I wanna jump out of a plane with a parachute . . . I wanna scuba dive properly . . . I wanna do the thing where you get dropped down into a cage and there’ll be sharks there . . . Like, I’ve been wanting to try erm, snails, for ages . . . I wanna go to cool places as well. Like, in Japan there’s a place called er, Suicide Forest . . . (Mason, 14, White)

Despite our seeking to explore the potential associations between vaping and the future use of tobacco and other substances, some participants articulated future imaginaries marked by progressively more expansive and daring experiences – swimming with sharks, Suicide Forest – many of which did not involve substance use at all. Likewise, other participants variously talked about futures in which they would become more ‘adventurous’, with travel, adult nightlife and risky leisure pursuits such as skydiving. The implicit sequentially connective logic employed in such future imaginaries is not simply between e-cigarettes and other drugs, but e-cigarettes and other experiences. Accordingly, such imagined futures are not marked by escalating drug use and progressive pharmacological dependence, but by greater self-determination through the fulfilment of personal ambitions, which pivot on adult *independence* in ways consistent with neo-liberal ideals of positive selfhood.

While narratives of self-determined experimentation were dominant in participants’ interviews in relation to vaping, smoking and other activities such as those outlined by Mason in the excerpt above, the possible outcomes of such experimentation – particularly the prospect of future addiction – were often considered a cause for concern. However, significantly, such concerns typically centred on an abhorrence of *dependence*, sometimes over and above the prospect of current or future ill-health:

I: What is it that worries you about . . . nicotine?

P: I don’t want that dependency. Like, I know people that smoke cigarettes where they *have* to have it, they are always smoking it.

I: It is not necessarily about it harming you? It’s about – you don’t want to be dependent?

R: If I was using it constantly, like, at a decent nicotine level and I stopped, obviously my body would be a bit, like, it would want it. But I seem to just . . . brush past it. (Joseph, 15, White)

In the follow-up interview, Joseph told us he did not have an ‘addictive mindset’ and repeatedly stressed that he was not dependent on vaping, but nonetheless needed to take care to avoid ‘abusing’ his e-cigarettes (like smokers who *have* to use cigarettes) for purposes other than those he deemed to be legitimate:

Say if I am working, then most of the time I will bring a bottle of liquid with me that is a bit higher in nicotine so like . . . I will use some [for] a bit of relaxing, I will just use that to relieve that stress. But I wouldn’t ever abuse it, it is just because . . . I will just put a little bit in, just to mix it up a bit, just so it is not all the same thing. (Joseph, 15, White)

Here, Joseph is, once again, describing a range of material–discursive practices: delineating between different kinds of (for him, legitimate) use – to relieve stress, to relax – through mixing liquids with different nicotine concentrations into his base vape. Material distinctions – blends and concentrations of nicotine liquids – entangle with discursive ones: ‘mixing it up’ *here* is okay, *there* it is not. ‘Abuse’ of nicotine – use without a purpose, or for a purpose other than those deemed legitimate – is considered unacceptable, and a potential marker of possible future dependence.

More generally, many participants emphasised concerns about nicotine dependence over and above the possible health consequences of inhaling vapour itself. Despite broader evidence on the relative risks of smoking and vaping publicised during the period of data collection, including Public Health England’s much-publicised finding that e-cigarettes are 95% safer than combustible tobacco (McNeill et al., 2015), numerous participants were *more* worried about vaping than smoking. For example, several dual e-cigarette and combustible tobacco users expressed concern that there was no tangible limit to how much one could vape compared with the finite length of a cigarette, and drew direct associations between frequency of vaping and degrees of ‘dependence’. Significantly, some dual users recounted switching from vaping back to smoking since the lower frequency of their cigarette smoking was seen to represent a reduced risk of addiction.

In such ways, cautionary tales presented in protectionist imaginaries of vaping futures – in particular, highly publicised concerns that vaping might be like, or even worse than, smoking – somewhat paradoxically served, in such cases, to steer usage trajectories towards individual tobacco pasts. Here ‘imagined futures’ can, again, be understood to have ‘real consequences’ for materialising futures-in-process, albeit in ways very different from those anticipated in such imaginaries.

Relatedly, particularly for those who had recently started vaping, using certain substances was often viewed in terms of fleeting, semi-detached phases involving tasting, testing, experiencing, but not committing to vaping and other substances/practices. Here, the imagined futures they articulated often described future selves who did not use any substances at all. Indeed, even for those more invested in vaping, an anticipated future non-dependent self was often understood as a vital prerequisite for use to be allowed to continue. Crucially, ‘dependency’ was not solely framed around pharmacological dependence:

I: [Has] the pattern to your vaping . . . changed or not? Do you tend to vape at particular times or particular events?

P: I think it has changed because . . . sometimes I would bring it to college . . . whereas now I’d leave it at home . . .

I: Why do you think that has changed?

P: I think it was more the fact that I wanted to keep it as something that I enjoy at home like, or something I enjoy separate rather than something that I am constantly [doing]. Especially as I use it socially as a tool as well, I don’t want it to become something l constantly rely on . . . Because I thought that would have stopped me from developing myself; you know I am relying on something. I am not kind of learning for myself or learning that social aspect for myself. (Aidan, 17, White)

Here, Aidan describes an array of material–discursive practices to avoid anticipated future dependency. However, again, the feared dependence is principally *social* dependence, whereby vaping would become indispensable as a ‘social tool’. ‘Constant reliance’ is to be avoided, and is implicitly treated as a marker of progressive dependence. Aidan is actively seeking to develop a future, more independent self, capable of ‘learning that social aspect’ unassisted by vaping. Implied in Aidan’s interview is how social/pharmacological dependence on his e-cigarette is understood to signify a possible future failed transition from adolescence towards an anticipated lack of competence in adulthood.

This excerpt, then, illustrates the simultaneous negotiation of three kinds of dependence/independence that articulate key themes across the data: imagined futures marked by pharmacological dependence, such as those expressed through gateway narratives; avoidance of social dependence, and an esteem for informed/knowledgeable independence and ‘healthy citizenship’; and the adoption of neo-liberal imaginaries of progressively independent *self*-management involving decreasing dependence on adults (e.g. parents). Significantly, in different ways, each runs directly counter to a dominant protectionist orientation characteristically adopted by those concerned with safeguarding young people, whereby adolescents are understood to lack the capacity to protect themselves from harms, while simultaneously having the greatest exposure to risk situations (Shivayogi, 2013).

### Dependence and Independence: ‘I Weren’t Even Determined’

Aidan’s concerns about possible future dependence both contain, and axiomatically respond to, core elements of the gateway/protectionist orientations towards youth vaping and the neo-liberal ideal of self-determination. These pivot on the understanding that substance use, particularly during adolescence, risks ‘derailing’ independent futures. Again, the prospect of failed transitions to adulthood – ‘going off the rails’, to continue with the same metaphor – and the prospect of futures characterised by escalating chemical (and, as Aidan articulates, social) dependence, figure prominently in such imaginaries. However, while our participants were, without exception, conversant with such ideas, several understood their vaping as integral to a series of investments towards independent, successful and accomplished futures – a means *of staying on the rails, not veering off them*.

Joseph, for example, consciously used vaping as a substitute for cannabis to avert a possible future of escalating substance use and the related ‘troubles’ he associated with this. Here, short-term pharmacological dependence on vaping (getting ‘hooked on the vape to get off the weed’) was, over his longer-term future-in-process, a means of attaining *independence*:

I: So when you say [you were] not smoking cigarettes, what were you smoking?

P: Cannabis. That’s it though, like nothing else . . . I had quite a bit of weed . . . And then I got into some trouble with the school and at home, so I thought I’ve gotta pack it in really. Before I got into doing it I, er, I had a vape, so I knew what it was like . . . So, I’ve just gradually stopped smoking it, and then like using the vape . . . And then I pushed it: at that time, I did put the nicotine in just so I could kind of get hooked on the vape to get off the weed. And then I just lowered it right back down and got it to zero, and then after a while I was like – I am not getting anything, so I just stopped it.

I : How did it feel doing that?

P: It just felt normal . . . I weren’t even determined; it was just like it was happening. I was just stopping, and then I didn’t. Even when I did try cannabis again, I didn’t even like it as such just because of all the stuff that came with it. Like getting in trouble. So, I just totally got off everything. (Joseph, 15, White)

Joseph goes on to explain that vaping provided a temporary basis for ongoing participation in a friendship group who regularly used cannabis and other substances that he no longer wanted to use. For Joseph the use of specific substances such as cannabis was inextricably bound up with other ‘stuff’ – trouble at home and school, participation within certain social groups and a possible future he wishes to avoid.

Joseph also alludes to his negotiation of a complex social landscape with attendant possible futures. His school draws intakes from one comparatively affluent region of Leicester, and two of the most economically deprived, diverse communities in the UK. Like others in our study, Joseph’s movement in and out of friendship groups and their related substance-using practices within and beyond his school simultaneously entailed the navigation of a differentiated nexus of gender, class and ethnicity-related ‘positions’ and related substance-using ‘dispositions’ in which unequal and economically deprived substance-using pasts continued to exert cultural and material constraints into prospective futures (Thirlway, 2018).

Joseph’s future-in-process thus entailed attempts to reconcile the remaining part of a social group in which trouble with authority and the use of cannabis and other drugs was a defining characteristic, with his desire to resist the possible futures that he associated with this group and its activities. For Joseph the ‘there’ of ‘harder’ drugs and potentially escalating pharmacological dependence is simultaneously one of social dependence and a sustained trajectory of adult disadvantage. Perhaps most significantly, this future imaginary was something Joseph consciously was investing *away from*, not *towards* through his use of e-cigarettes. Vaping was approached then, not as a disruption to, or a derailing of, Joseph’s independent adult future development, but as a means to achieve this and to escape the enduring influence of materially deprived pasts.

This material–discursive enmeshing of vaping practices – the entangled nexus of, for example, mixing liquids and mixing in social groups – is a common theme across our data. In the following, Evie describes how vaping forms a core axis of mutual engagement, serving as a basis for participation in friendships to the extent that its primary value is social, over and above the material practice of ‘vaping itself’:

I: Do you think if everyone just suddenly stopped, you’d be done too then?

P: Yeah, to be honest I wouldn’t miss it, it’s not like a regular part of my life . . . Like, I don’t mind. It’s not . . . about the vaping itself . . . it just gives you all something to have: like a common ground, and those that aren’t involved in it can still get involved in it. Like, you know, if we all decided we want to go down to the cinema and three friends didn’t have the money, then it would be difficult to do that. But if three friends don’t have vapes and eight of us do, then we can all share and it’s no problem. (Evie, 17, White)

As extracts from Evie and other participants show, imagined futures are not solely imagined, they are emergent and contingent futures-in-process: partly invested and shaped through the conditions of their own possibility, sometimes in ways that follow very different directions from those anticipated in such imaginaries. Such investments involve a panoply of material-discursive practices: cognitive/affective intrapersonal investments – changes to vaping practices, or abstaining from e-cigarette use to avert social and/or pharmacological dependence; interpersonal investments, investing in particular friendship networks, vaping for inclusivity – to allow oneself and others to ‘stay in’ with a friendship network in which substance use is central to common repertoires of practice and engagement, mutual identifications, peer connections.

As discussed, such investments allude to a range of social and material conditions across our sample – from aspirations to visit Japan, to mixing in groups where the consumption of a range of drugs is commonplace at a young age, and where material affordability is a central consideration – reflecting the diverse composition of our sample. As a wealth of literature attests, the likelihood of pursuing pathways that lead towards drug-dependent futures is not simply a matter of individual choices, decisions and aspirations, and related ‘gateway effects’; it is linked to multiple axes of material deprivation and social disadvantage (e.g. Pilkington, 2007). Indeed, under particular social conditions, seemingly ‘irrational’ behaviours such as substance use can be understood as quite ‘rational’ coping mechanisms for those enmeshed in ‘irrational’ life circumstances. Moreover, the uptake of vaping, which to outward appearances might be deemed a ‘maladaptive’ behaviour, can, when viewed in context, be understood to form part and parcel of young people seeking ways out of culturally and chemically circumscribing futures based in pasts that entail a complex entanglement of substance use and social disadvantage.

Indeed, a move towards using e-cigarettes to *avert* disadvantaged and dependent futures found a multitude of expressions in our study. For most participants, the use of vaping experimentally, recreationally or performatively – particularly when employed as a display behaviour to peers – was held repeatedly to characterise earlier phases of young users’ vaping. By contrast, those who ‘stayed with’ vaping would typically understand themselves as having transitioned towards more ‘pharmacological’ use; including using e-cigarettes to combat stresses related to the pressures of adolescence, in particular exams:

I know it’s bad and I know I definitely shouldn’t be doing it, but . . . I am young now, I can probably try and quit later on down the line. There’s no point trying to give up something that is going to make me more stressed. Like trying to give it up while I am so stressed about my exams. (Poppy, 17, White)

And then I came home one day, and my dad was like ‘Empty your pockets’. . . . I was like ‘I am using it for a positive thing’ . . . they were annoyed at the time . . . it is mostly just family telling me ‘you have got to get school done’ and all that, and ‘you can say what you like, we won’t condone it; it is your life, but you have to get these GCSEs’. (Zev, 14, South Asian)

Poppy’s extract typifies accounts of more established, invested e-cigarette users in our study. It expresses a kind of future-in-process trade-off: *I will continue to vape now so that I can secure a better, credentialised future* (in which I will likely no longer vape). Zev’s remarks also illustrate, somewhat ironically, how young people might seemingly push back against the very adults with whom they are seeking to comply (i.e. parents and teachers) to attain their educational futures. Rather than expressing the solely recreational, dependence-driven substance use assumed by protectionist/gateways future imaginaries, our participants described using e-cigarettes as part of a panoply of resources in the pursuit of self-actualisation. In the futures-in-process of some young users, e-cigarettes come to be treated instrumentally, managing stress in the *here and now* to enable success in exams and thereby invest in successful, hoped for, aspirational futures in the *there and then*.

Taken together such examples challenge the framing of substance use in young people as *inevitably* a derailment of efforts to achieve successful adulthood, and require instead a consideration of the conditions under which substance use is the partial means to pursue, and possibly secure, futures that are mutually hoped for and worked towards by young people and those responsible for them.

## Conclusion

Our discussion above has entailed a synthetic consideration of three areas of concern: futures and temporality; gateways and e-cigarettes; and adolescence and late-modernity. By bringing these conceptual and empirical concerns into dialogue, we have sought to highlight the fundamental interdependence of *present futures* and *future presents* (Adam, 2023). To do so, we have advanced the concept of *futures-in-process* to capture an array of material–discursive practices that entail concurrent processes of future orientation and future making. Here, we refer to the dynamic interplay between the biographical futures of young vapers and the related generational futures of gateway imaginaries. We have also demonstrated how gateway concerns regarding young people’s e-cigarette use involve ‘retrospective anticipatory’ framings: how imaginaries of young vaping futures are based in smoking pasts and ‘past futures’. Accordingly, we have shown how tobacco pasts have come to have an enduring influence over vaping futures, discursively-materially conditioning e-cigarettes as ‘tobacco products’, fuelling heightened concern and uncertainty regarding the relative/future risks of smoking and vaping, and informing the practices of policy makers, teachers and parents, and of young users themselves.

Following from this, we have argued that gateway imaginaries of vaping leading to tobacco (and other drugs), and as *like smoking*, have real consequences. Yet, these can be realised in the materialising futures-in-process of young vapers in ways profoundly different from those anticipated by gateway thinking. Participants whose use does not develop beyond the understanding that e-cigarettes are things to be tried – like loom bands or fidget spinners (Hughes et al., 2021; Tokle, 2020) – tended to remain within a phase in which potentially more harmful substances (e.g. tobacco, cannabis, cocaine) were still ‘on the menu’. Consequently, the route towards, and possible connection between, different substances and experiences, was characteristically more one of *experimentation* than *escalation*. Thus, contra the imagined futures of gateway thinking, the use of more harmful drugs and potential escalations of physical dependence resided not so much at the ‘end of the road’, but somewhere towards its beginning. Starting ‘here’ did not necessarily take young people ‘there’. In fact, more regular, and ‘invested’ vaping sometimes marked important steps to *avoid* the ‘there’ of gateway future imaginaries, especially futures marked by increased dependency. Moving beyond a phase of e-cigarette use characterised by playful experimentation, including with other (harder) drugs, typically involved young people making material, cognitive, social and emotional investments in vaping. Accordingly, what may appear ostensibly to be a mark of progressive dependence and a transition towards the ‘there’ (escalating harm) of gateway future imaginaries – that is, continued, regular, more stable patterns of e-cigarette use – may instead mark a move away from this, *despite* materially circumscribed conditions that might otherwise steer them that way.

Vaping futures-in-process, then, are both *produced and constrained*, not simply through progressive drug dependence, but by a complex array of material–discursive *techniques of futuring*, including reflexive investments that are shaped in and through the conditions of their own possibility. Such investments entail an interplay of social positions and substance-using dispositions where participation in certain friendship networks, for example, might simultaneously entail other kinds of investment: in certain practices, substances, affective identifications and, in some cases, strategies to avoid enduring disadvantaged pasts and the possible futures that relate to those.

In this way, our analyses radically challenge an axiomatic framing in popular, scientific and policy discourse, which anticipates substance use by young people as invariably entailing the *derailment* and not *pursuit* of esteemed self-determined futures. Departing from this anticipatory framing has major implications for how we conceptualise and research young people’s substance use. It suggests the need for a shift in policy and scientific research away from a preoccupation with whether the consequential narratives contained in gateway thinking might, or might not, be true in some temporally correlative sense, and towards a consideration of how contrasting imaginations of the future are materialising in biographical and generational futures-in-process. By employing such an approach here in relation to youth vaping, we have shown how developing pathways of substance use can entail a series of investments away from the future harms anticipated by gateway thinking and neo-liberal imaginaries of failed adolescence, rather than what might otherwise be imagined as volitionary divestments arising from escalating substance dependency and failed selfhood. Finally, we propose that the example of youth vaping serves also to demonstrate how the processes by which futures come to materialise might be theoretically and empirically apprehended in a way that avoids a sociological tendency to separate considerations of ‘futures thinking’ and ‘futures making’ by exploring how these are fundamentally interrelated aspects of futures-in-process.

## Notes

1. We now know of course that tobacco was and is anything but innocuous or ‘innocent’ in the sense of being unharmful. However, during this period, to continue the legal metaphor, *the verdict was still out* on tobacco, with a range of high-profile opponents – King James I among them – who warned against its *abuse* as a recreational ‘vice’, and many influential exponents, including leading physicians of the time, who extolled the virtues of its ‘temperant’ *use* as a medicinal remedy (see, for a discussion of these issues, Hughes, 2003).

## References

[bibr1-00380385241290745] Adam B (2004) Towards a new sociology of the future. Cardiff University. Unpublished paper. Available at: http://www.cf.ac.uk/socsi/futures/newsociologyofthefuture.pdf

[bibr2-00380385241290745] Adam B (2011) Wendell Bell and the sociology of the future: Challenges past, present and future. Futures 43(6): 590–595.

[bibr3-00380385241290745] Adam B (2023) Futures imperfect: A reflection on challenges. Sociology 57(2): 279–287.

[bibr4-00380385241290745] Alexander FW (1930) Tobacco: Discovery, origin of name, pipes, the smoking habit and its psychotherapy. The Medical Press 181: 89–93.

[bibr5-00380385241290745] ASH (2023) Use of E-Cigarettes (Vapes) Among Young People in Great Britain. Available at: https://ash.org.uk/uploads/Use-of-vapes-among-young-people-GB-2023-v2.pdf?v=1697209531 (accessed October 2023).

[bibr6-00380385241290745] Beck U (1992) Risk Society: Towards a New Modernity. London: Sage.

[bibr7-00380385241290745] Bell K Keane H (2014) All gates lead to smoking: The ‘gateway theory’, e-cigarettes and the remaking of nicotine. Social Science & Medicine 119: 45–52. 25150650 10.1016/j.socscimed.2014.08.016

[bibr8-00380385241290745] Burton R (1638) The Anatomy of Melancholy. Oxford: Henry Cripps.

[bibr9-00380385241290745] Galderisi A Ferraro VA Caserotti M , et al (2020) Protecting youth from the vaping epidemic. Pediatric Allergy and Immunology 31: 66–68. 10.1111/pai.13348 33236441

[bibr10-00380385241290745] Giddens A (1991) Modernity and Self-Identity. Cambridge: Polity Press.

[bibr11-00380385241290745] Gokmenoglu B (2022) Temporality in the social sciences: New directions for a political sociology of time. The British Journal of Sociology 73(3): 643–653. 35325469 10.1111/1468-4446.12938 PMC9314931

[bibr12-00380385241290745] Hajer M Pelzer P (2018) 2050 – An energetic odyssey: Understanding ‘techniques of futuring’ in the transition towards renewable energy. Energy Research & Social Science 44: 222–231.

[bibr13-00380385241290745] Halford S Southerton D (2023) What future for the sociology of futures? Visions, concepts, and methods. Sociology 57(2): 263–278.

[bibr14-00380385241290745] Hitchman S Brose LS Brown J , et al. (2015) Associations between e-cigarette type, frequency of use, and quitting smoking: findings from a longitudinal online panel survey in Great Britain. Nicotine & Tobacco Research 17(10): 1187–1194. 25896067 10.1093/ntr/ntv078 PMC4580313

[bibr15-00380385241290745] Hughes J (2003) Learning to Smoke: Tobacco Use in the West. Chicago: University of Chicago Press.

[bibr16-00380385241290745] Hughes J Sykes K Hughes K , et al (2021) From gateways to multilinear connections: A qualitative longitudinal investigation of the relationships between vaping and smoking among adolescent users. International Journal of Drug Policy 92: 103341. 10.1016/j.drugpo.2021.103341 34229192

[bibr17-00380385241290745] Hughes K Hughes J Tarrant A (2022) Working at a remove: Continuous, collective, and configurative approaches to qualitative secondary analysis. Qual Quant 56: 375–394. 33746296 10.1007/s11135-021-01105-x PMC7960881

[bibr18-00380385241290745] Jordt SE (2023) Synthetic nicotine has arrived. Tobacco Control 32: e113–e117. 10.1136/tobaccocontrol-2021-056626 PMC8898991 34493630

[bibr19-00380385241290745] King B Jones C Baldwin G , et al (2020) The EVALI and youth vaping epidemics: Implications for public health. New England Journal of Medicine 382(8): 689–691. 31951683 10.1056/NEJMp1916171 PMC7122126

[bibr20-00380385241290745] Leicester City Council (2019) Indices of deprivation 2019: Briefing on implications for leicester. Division of public health. Leicester city council. Available at: https://www.leicester.gov.uk/media/x33jvoab/indices-of-deprivation-in-leicester-briefing-on-implications-2019.pdf (accessed October 2023).

[bibr21-00380385241290745] McNeill A Brose L Calder R , et al (2015) E-cigarettes: An evidence update. Public Health England. Available at: https://assets.publishing.service.gov.uk/media/5b6c3f57ed915d30f140f822/Ecigarettes_an_evidence_update_A_report_commissioned_by_Public_Health_England_FINAL.pdf (accessed October 2023).

[bibr22-00380385241290745] Munafò M (2019) Are e-cigarettes tobacco products? Nicotine and Tobacco Research 21(3): 267–267. 29931122 10.1093/ntr/nty130

[bibr23-00380385241290745] Nkansah-Amankra S (2020) Revisiting the association between “gateway hypothesis” of early drug use and drug use progression: A cohort analysis of peer influences on drug use progression among a population cohort. Substance Use & Misuse 55(6): 998–1007. 32077787 10.1080/10826084.2020.1720245

[bibr24-00380385241290745] Oomen J Hoffman J Hajer M (2022) Techniques of futuring: On how imagined futures become socially performative. European Journal of Social Theory 25(2): 252–270.

[bibr25-00380385241290745] Pierce J Chen R Leas E , et al (2021) Use of e-cigarettes and other tobacco products and progression to daily cigarette smoking. Pediatrics 147(2): e2020025122. 10.1542/peds.2020-025122 PMC7849197 33431589

[bibr26-00380385241290745] Pilkington H (2007) Beyond ‘peer pressure’: Rethinking drug use and ‘youth culture’. International Journal of Drug Policy 18(3): 213–224. 17689368 10.1016/j.drugpo.2006.08.003

[bibr27-00380385241290745] Shivayogi P (2013) Vulnerable population and methods for their safeguard. Perspect Clinical Research 4(1): 53–57. 10.4103/2229-3485.106389 PMC3601707 23533983

[bibr28-00380385241290745] Steinberg L (2010) A dual systems model of adolescent risk-taking. Developmental Psychobiology 52(3): 216–24. 10.1002/dev.20445 20213754

[bibr29-00380385241290745] Taylor C (2003) Modern Social Imaginaries. Durham, NC: Duke University Press.

[bibr30-00380385241290745] Thirlway F (2018) How will e-cigarettes affect health inequalities? Applying Bourdieu to smoking and cessation. International Journal of Drug Policy 54: 99–104. 29414491 10.1016/j.drugpo.2018.01.009 PMC5912796

[bibr31-00380385241290745] Thomas WI Thomas DS (1928) The Child in America: Behavior Problems and Programs, 2nd edn. New York, NY: Alfred A. Knopf.

[bibr32-00380385241290745] Tokle R (2020) Vaping and fidget-spinners: A qualitative, longitudinal study of e-cigarettes in adolescence. International Journal of Drug Policy 82: 102791. 32516687 10.1016/j.drugpo.2020.102791

[bibr33-00380385241290745] Tutton R (2017) Wicked futures: Meaning, matter and the sociology of the future. The Sociological Review 65(3): 478–492.

